# Factors influencing the implementation of Home-Based Stroke Rehabilitation: Professionals’ perspective

**DOI:** 10.1371/journal.pone.0220226

**Published:** 2019-07-25

**Authors:** Dinja J. van der Veen, Carola M. E. Döpp, Petra C. Siemonsma, Maria W. G. Nijhuis-van der Sanden, Bert J. M. de Swart, Esther M. Steultjens

**Affiliations:** 1 Institute of Health Studies, University of Applied Sciences HAN, Nijmegen, the Netherlands; 2 Healthy Living, TNO Leiden, Leiden, the Netherlands; 3 University of Applied Sciences THIM, Nieuwegein, the Netherlands; 4 Faculty of Health, University of Applied Sciences Leiden, Leiden, the Netherlands; 5 Research Institute for Health Sciences, IQ healthcare, Radboud University Medical Center, Nijmegen, the Netherlands; 6 Department of Neurorehabilitation, Radboud University Medical Center, Nijmegen, the Netherlands; Università degli Studi di Perugia, ITALY

## Abstract

**Background:**

Stroke has a major impact on survivors and their social environment. Care delivery is advocated to become more client-centered and home-based because of their positive impact on client outcomes. The objective of this study was to explore professionals’ perspectives on the provision of Home-Based Stroke Rehabilitation (HBSR) in the Netherlands and on the barriers and facilitators influencing the implementation of HBSR in daily practice.

**Methods:**

Semi-structured focus groups were conducted to explore the perspectives of health and social care professionals involved in stroke rehabilitation. Directed content analysis was performed to analyze the transcripts of recorded conversations.

**Results:**

Fourteen professionals participated in focus groups (n = 12) or, if unable to attend, an interview (n = 2). Participants varied in professional backgrounds and roles in treating Dutch clients post stroke. Barriers and facilitators influencing the implementation of HBSR in daily practice were identified in relation to: the innovation, the user, the organization and the socio-political context. Participants reported that HBSR can be efficient and effective to most clients because it facilitates client- and caregiver-centered rehabilitation within the clients’ own environment. However, barriers in implementing HBSR were perceived in a lack of (structured) inter-professional collaboration and the transparency of expertise of primary care professionals. Also, the current financial structures for HBSR in the Netherlands are viewed as inappropriate.

**Discussion:**

In line with previous studies, we found that HBSR is recognized by professionals as a promising alternative to institution-based rehabilitation for clients with sufficient capabilities (e.g. their own health and informal support).

**Conclusion:**

Multiple factors influencing the implementation of HBSR were identified. Our study suggests that, in order to implement HBSR in daily practice, region specific implementation strategies need to be developed. We recommend developing strategies concerning: organized and coordinated inter-professional collaboration, transparency of the expertise of primary care professionals, and the financial structures of HBSR.

## Introduction

Stroke is one of the major causes of mortality, loss of independence, and lower quality of life of stroke survivors and has a great impact on the social environment [1]. Between 2010 and 2030 the absolute number of people with a stroke is expected to increase by 56% in men and 37% in women [2]. Also, stroke is known to have major socio-economic consequences. The financial burden placed on European countries by stroke is huge. For 2010, the estimated cost of stroke in Europe was €64 billion [3].

### Stroke rehabilitation in the Netherlands

In the Netherlands, stroke rehabilitation is organized and delivered in various ways. From the late ‘90, three main types of stroke rehabilitation can be distinguished in the Netherlands.

Firstly, stroke rehabilitation can be offered as institution-based rehabilitation: organized within hospitals, rehabilitation centers and nursing homes. Within institution-based rehabilitation, care is centered around a diagnosis. Professionals are specialized in treating clients with this specific diagnosis. Also, within the institution, regular (formal and informal) inter-professional meetings take place [4].

Secondly, stroke rehabilitation can be offered on outpatient basis. After their transfer home (from the stroke unit or institution-based rehabilitation), stroke survivors can consult outpatient rehabilitation professionals. Stroke survivors receiving outpatient rehabilitation live at home and visit the institution to receive therapy.

Thirdly, stroke rehabilitation can be offered as Home-Based Stroke Rehabilitation (HBSR). During HBSR (Home-Based Stroke Rehabilitation) rehabilitation is offered within the home environment of the client. It includes community-based rehabilitation delivered by primary care professionals, such as occupational therapists, physical therapists, speech therapists, dieticians, social workers, nurses and general practitioners [5]. A broad range of professionals can be involved during HBSR, because the impact of stroke is multifaceted, affecting a broad range of body functions, activities and participation patterns [6]. In the Netherlands primary care is not nationally organized: professionals deliver care from independent private practices and from a variety of institutions. General health insurances cover a certain (predefined) amount of treatment hours for selected disciplines only. The variety in financial legislations between these selected disciplines is large. Sometimes additional treatment hours and/or disciplines are financed, depending on the severity of symptoms, personal circumstances and insurance coverage. Insurance coverage differs per person and depends on the selection of optional insurances.

### Home-Based Stroke Rehabilitation (HBSR)

Healthcare professionals and organizations are challenged to provide high quality health and social care, in a client centered and cost-efficient manner. To improve the quality and efficiency of care, the location of care delivery is shifting from institution-based settings to home-based services such as HBSR.

HBSR is known for its positive impact on client outcomes. HBSR resulted in more independent clients [7, 8] who are better at performing daily activities [8, 9] and who are more satisfied with their treatment compared to clients who receive conventional (institution-based) rehabilitation [8–12]. Also, HBSR is shown to reduce the length of hospital stay and to decrease the likelihood of admittance in a long-term stay facility [8]. Furthermore, HBSR has the benefit of treating clients within a familiar environment. According to prior studies this tends to stimulate mental and physical activity, provides more meaning to tasks [13, 14] and prevents potential problems with the transfer of learned skills from the training setting to executing daily activities [15].

### Implementing HBSR

In the Netherlands a number of reforms and new policies have been implemented over the last years to facilitate client-centered and cost-effective care. These changes include policies increasing the responsibility of the municipalities for care and welfare on the municipality and transferring more responsibilities from professional carers to civilians and local communities themselves [16]. Despite these efforts, the client-centered and cost-effective provision of high quality care remains a challenge because guidelines, practical suggestions and organisational support seems to be missing [17]. Consequently, both researchers as well as healthcare professionals initiate new regional projects [17–19]. According to the literature, this does not only take place in the Netherlands. Many clients do not receive appropriate care, or receive unnecessary or even harmful care [20].

Major difficulties can arise when implementing innovations, like HBSR, into routine practice. Even though previous studies have shown positive effects of HBSR [7–15], innovations are not always provided to those clients for whom it could be beneficial [21]. Prior studies show that clients and caregivers experience a gap after institution-based rehabilitation (e.g. delays and discontinuity of therapy and feeling abandoned and unsupported) and poor accessibility of community services [22, 23]. In order to further implement an innovation like HBSR, context specific implementation strategies are needed at different levels [24–26].

This Dutch study explores and describes professionals’ perspectives on determinants that could influence the further implementation of HBSR. These insights can guide the selection of context specific implementation strategies. This study will not only provide insight into region specific factors influencing implementation, but also general issues playing a role in the implementation of HBSR.

In this qualitative focus group study we focused on the following questions:

1. How do professionals characterize stroke rehabilitation services that are currently provided in the Netherlands?
2. What are the current and potential barriers and facilitators influencing the implementation of HBSR in their daily practice, according to professionals?

## Methods

### Study design and participants

Two focus groups were conducted in November and December of 2013, utilizing a naturalistic study design [27] based upon a constructionist epistemology for the study’s exploratory aims. Constructionist epistemology have theorized that reality is socially constructed [28, 29]. We wanted to explore professionals’ perspectives on elements that could influence the implementation of HBSR in the future. We anticipated that the interaction between participants during focus groups could maximize the exploration of different perspectives to construct ‘reality’ and would lead to more in-depth insights than individual interviews.

As mentioned in the introduction, a broad range of professionals can be involved in HBSR. In order to include a wide range of perspectives, professionals who were invited to participate were: 1) working in different care environments (primary, outpatient and institution-based care), 2) were experienced in treating clients after stroke, and 3) had different roles (advisor, therapist, specialist and manager) within the rehabilitation process.

Considering the wide range of professional’s perspectives, the presence of a focus group moderator, setting an informal atmosphere and asking to elaborate on statements and/or topics related to the key questions, was considered essential [30]. Individual interviews (using the same interview guide) were considered an alternative when a specific discipline was unable to take part in the focus groups.

According to the guidelines of the local ethical committee of the HAN University of Applied Science, our research was conducted conform the Declaration of Helsinki [31]. Therefore the research project could be conducted without approval of a research ethics committee.

### Recruitment and sampling

A convenience sample was used. Contact information of professionals known to the broad national network of two of the authors (ES and BS) were gathered. Professionals were selected if they represented one of the professions that are most frequently involved treating clients after stroke and if they were known to be interested in HBSR. The first author (DV) contacted the professionals by e-mail and informed them about the subject under study and the research questions. Thereafter professionals were asked to participate in a focus group to discuss their point of view with other professionals in relation to the research questions. On request invited professionals were contacted by phone to give additional information about the study. Professionals who were unable to participate in the study were asked to recommend a colleague who might be able to participate (respondent driven sampling). These professionals were thereafter informed and invited by the first author (DV).

### Data collection procedure

Data were collected during semi-structured focus groups (or interviews). During the focus groups, efforts were made to set an informal atmosphere. Participants were welcomed with drinks and snacks and names (but not their professional backgrounds) were displayed in front of each participant. After each participant introducing themselves, the focus group moderator (CD) made explicit that different perspectives were expected and welcomed, and that everyone’s viewpoint was highly valued. Data collection started with asking participants to give verbal informed consent to audiotape the interviews.

An interview guide with nondirective, open-ended questions was used [32]. The first questions were aimed at defining HBSR. To get the participants ‘warmed-up’, open-ended questions were directed on gaining insight in the participants’ perceptions and experiences regarding the current status of stroke rehabilitation in the Netherlands. Thereafter the interview guide contained open-ended questions relating to the participants’ opinions about factors influencing the implementation of HBSR within their daily practice. These key questions focused on determinants influencing the implementation of HBSR, as stated in a previous study (i.e. the innovation, the users, the organization and social-political context) [21, 26].

The focus groups were moderated by one of the authors (CD), who has extensive knowledge of HBSR and is experienced in moderating focus group discussions. If necessary, the focus group moderator (CD) asked participants to elaborate on their statements and/or on specific topics related to the key questions (determinants influencing implementation as stated in previous study [21]).

During the focus groups a secretary (DV) was present to take notes. When information was collected on all key questions and no new information emerged, the secretary provided a brief summary of the discussion for verification purposes. Thereafter the focus group moderator asked if anything was missed.

### Data analysis

The focus groups and interview recordings were transcribed verbatim by the first author (DV). In the transcript each participant was assigned an ID number, so that statements from participants can be distinguished when reporting results, without using the names of participants.

To identify key issues and patterns in the data, the first author (DV) performed content analyses based on the directed content analysis described by Hsieh & Shannon [32] (using Atlas.ti 7.5.2). Raw data were analyzed through a line-by-line review of the transcripts to identify quotes. Quotes relating to experiences with the current status of stroke rehabilitation in the Netherlands and quotes relating to factors influencing the implementation of HBSR were highlighted. The highlighted quotes were coded through open coding (DV), i.e. no predetermined codes were used [32]. Thereafter, the codes referring to the same key issue were grouped, resulting in categories emerging from the data. These categories were grouped using determinants stated in a previous study [21]: the innovation, the users, the organization and social-political context [21]. This concludes the last stage of the directed content analysis [32]. Consensus on codes and categorization was reached through discussion between two of the authors (DV, ES). After reaching consensus, the final categorization of codes was reviewed by two other authors (CD, PS).

## Results

### Participants

A total of 15 professionals were willing to participate in the study. Most participants did not know each other, and none of the participants knew the focus group moderator (CD) and the primary researcher (DV). No contact was established with speech therapists working within primary care, despite multiple efforts trying to get in touch by e-mailing potential participants. However, the participating case manager could provide this perspective because she worked as a primary care speech therapist until recently.

The participating professionals were split into two focus groups. Groups were created to reflect a variety in disciplines, gender, age, workplace setting and years of experience [30]. Three professionals could not attend any of the focus groups (a geriatrician, a general practitioner and a rehabilitation physician). These professionals agreed to take part in individual telephone interviews. Unfortunately, the interview with the rehabilitation physician could not be executed within the timeframe of the study due to the tight work schedule of the physician.

Participants were based in the southeastern, southern and western regions of the Netherlands as occupational therapists (n = 2), physical therapists (n = 2), case managers (n = 2), a psychologist, an elderly advisor, a nurse, a speech therapist, a nursing home manager, a manager in allied healthcare, a general practitioner and a geriatrician. The group of participants consisted of five men and nine women between the ages of 28 to 61. Participants’ work settings included hospitals, nursing homes (delivering primary care), home care organizations, a primary care rehabilitation and healthcare center, an outpatient facility and a general practitioners’ office.

### Participants’ perspectives on the current status of stroke rehabilitation in the Netherlands

Participants gave their perspectives on how stroke rehabilitation is organized and delivered in the Netherlands, distinguishing 1) institution-based rehabilitation, 2) outpatient rehabilitation and 3) HBSR. According to the participants, the form of rehabilitation that is most suitable for a specific client depends on multiple factors (e.g. clients’ situation, client system and resources). However, it is not clear what the exact clinical criteria (and cut-off points) are.

Firstly, participants stated that intensive inter-professional collaboration characterizes institution-based rehabilitation. According to the participants the regular (formal and informal) inter-professional meetings facilitate inter-professional collaboration. However, participants reported that institution-based rehabilitation is delivered to less people and for shorter durations. The lower (cost) effectiveness compared to other types of rehabilitation were stated as the main reason for this.

Relating to outpatient rehabilitation, participants stated that especially the cognitive outpatient clinic is consulted by a lot of clients after stroke. Participants reported that these clients experience difficulties performing activities of daily living in their own environment after institution-based rehabilitation.

According to participants an important characterization of HBSR is the amount of financed treatment hours. Participants reported that the amount of guidance and treatment they can offer differs, depending mostly on insurance coverage of the client. Participants also stated that HBSR is highly valued as a safety net after discharge. Clients with neurological difficulties can experience paramount problems in their home environment because of (1) the large gap between the institution and the home situation, (2) problems generalizing and transferring learned skills and (3) the rehabilitation setting hindering exposure (and recovery) of cognitive difficulties. It was suggested HBSR could embody client-centered follow-up care after discharge, coordinated by a “key agent”.

### Determinants influencing the implementation of HBSR in the Netherlands

Participants reported their perspectives on determinants influencing the further implementation of HBSR, as stated in a previous study (i.e. the innovation, the users, the organization and social-political context) [21]. An overview can be found in S1 Table.

#### Determinants of the innovation: Pros and cons of HBSR

Focus group participants reported the importance of client-centered treatment, tailored to the clients’ and caregivers’ needs. Participants characterized institution-based treatment to be standardized, leaving little room for client-centered treatment and caregivers involvement. Meanwhile, participants reported that the less rigid structure of HBSR makes it easier to adapt the rehabilitation process to the needs and preferences of clients and caregivers.

Also, participants reported some advantages of treating clients within their own environment. One of them concerns the professional being a guest in the clients’ home. According to participants this can strengthen the client’s position. Participants experience this can facilitate a stronger voice of clients and caregivers in reporting their needs and wishes regarding the rehabilitation process.

Moreover, treating clients in their own environment has some other consequences. Participants stated benefits of overcoming problems with generalizing and transferring learned skills from the training setting to the home setting. Participants stated that there currently is a large gap between the institution (a structured and low-stimulus environment) and the home setting (a high-stimulus environment where people need to build up and follow their own daily structure).

*“The ‘body function-oriented’ institution-based rehabilitation*, *combined with the constructed environment*, *covers up most of the client’s cognitive problems*. *So when a client returns home*, *they are up for a challenge*. *Guaranteed*.*”*

(neuropsychologist, outpatient rehabilitation)

Participants also mentioned that everyday living experiences in the home environment can facilitate meaningful task specific training “around the clock”. Participant stated this could make the treatment more effective.

*“Every activity can be a challenge*. *Someone* <client> *has a lot to do*. *They find themselves within a skills lab*, *for 16 hours a day*!*”*

(manager in allied healthcare, primary care)

According to participants, especially clients with higher (physical and mental) capabilities can benefit from the higher intensity of HBSR. On the other hand, according to participants, this higher intensity of treatment could make HBSR more demanding to clients compared to institution-based rehabilitation. Participants reported that this, combined with the lower amount of financed supervised training and medical care associated with HBSR, could make HBSR less suitable to clients with lower capabilities and those in frail health.

#### Determinants of the user: Client and caregiver characteristics

The participants emphasized the large heterogeneity regarding the healthcare needs of clients post stroke. According to participants the main reason for this heterogeneity is the large variety in cognitive deficits after stroke and the impact on daily life. However, as mentioned above, institution-based treatment after stroke is experienced to be standardized and therefore not always in line with the specific clients’ needs.

*“The complexity*, *the reported needs*… *they are so diverse*. *However*, *hospital care is standardized; everything that needs to be done is described per second*.*”*

(case manager 1, primary care)

Participants also emphasized the important role caregivers can have within the rehabilitation process of people after stroke. According to participants it is important to inform, instruct and coach caregivers, in order to prepare them for the client’s transfer home. This could limit the caregiver’s burden and preserve or expand the client’s capabilities. However, participants experience that the involvement of primary caregivers is limited during institution-based rehabilitation.

*“They <*client and caregiver> *hear*: *“It was a stroke”*, *and the next second they are home again*. *Caregivers are not included at all*.*”*

(case manager 1, primary care)

#### Determinants of the user: Professional expertise

Participants reported the importance for sufficient expertise in order to deliver care to people after stroke. According to participants expertise can be built by being educated in the field of neurology and treating clients after stroke on a regular basis. However, participants experience that not all professionals meet these conditions. Participants stated that the expertise of primary care professionals is diverse and sometimes lacking.

“*How can you build expertise when you treat three to four stroke clients a year?*”

(physical therapist, institution-based rehabilitation)

Participants not only experience the amount of expertise within primary care to be diverse, but also to be not transparent. Participants reported that it is unclear who has the required (and/or the most) expertise concerning stroke rehabilitation and an easily accessible overview of (local) professional is lacking. Not knowing who has sufficient or the most expertise makes it difficult for clients, caregivers, and referring professionals to know which professional they should consult for HBSR.

*“When a client needs HBSR*, *I do not know to which professional I need to refer him to*. *Everybody* <professionals> *says they can deliver the treatment*, *but I do not know if they really can and who is the best*.*”*

(general practitioner, primary care)

Participants reported that the diversity in the expertise of primary care professionals, and this expertise not being visible, makes it harder to collaborate. Participants stated it is not easy to involve other professionals for inter-professional collaboration, when you do not know who to consult.

However, not only the expertise of professionals is experienced to influence collaboration. According to focus group participants some organizational factors influence the ability for inter-professional collaboration as well.

#### Determinants of organization: Transparency in working together

According to participants, inter-professional collaboration is needed to deliver good care. Participants reported the need for a comprehensive treatment plan and regular meetings to make explicit “the overall goal” and “who does what”.

However, participants experience that within primary care, most (if not all) disciplines have their own method, ‘language’ and way of documenting treatment findings into health records. Also, the absence of regular scheduled meetings within primary care is experienced to hinder collaboration. Within institution-based care these meetings take place on a regular basis. According to participants it takes a lot of effort to organize these meetings within primary care because central organization and financial support are lacking.

*“Local authorities are also developing their own documentation system*. *Some bits are new*. *Anyway*, *it is yet another new system”*.

(case manager 2, primary care)

*“Within primary care professionals simply do not run into each other*. *We need to organize inter-professional contact to be able to collaborate”*.

(manager in allied healthcare, primary care)

Beside a lack of organized collaboration, participants also experience a lack of *(central) coordination* of community care. Participants mentioned that there should be one “key agent”, who facilitates the client during the different stages of rehabilitation. According to participants, this person should coordinate care delivery, stand beside the client and should know where and how to find and access professionals with sufficient expertise.

*“Everybody holds a piece of the elephant*, *but nobody sees the whole picture”*

(neuropsychologist, outpatient rehabilitation)

A lack of collaboration within primary care is experienced to be influenced by the large number of *different professionals* involved in primary care, while “trust” and “knowing each other” are supporting factors for cooperation between professionals.

#### Determinants of the social-political context: Financing structure

Within the Dutch healthcare system, the involved disciplines and the amount of financed treatment hours after stroke are predefined and restricted per discipline. The last decades many changes have been made concerning the financing of rehabilitation after stroke in the Netherlands.

Participants experience the predefined and restricted character of financing rehabilitation after stroke per discipline to be inappropriate considering the heterogeneity among clients. Participants reported the need for a more client-centered way of financing rehabilitation after stroke.

*“One of our clients was in need for intensive speech therapy*, *but otherwise was ready to go home*. *However*, *because the sufficient amount of speech therapy was not financed within primary care*, *the client needed to stay*. *Such a shame*…*”*

(speech therapist, institution-based rehabilitation)

Participants suggested the implementation of a financial structure depending on the clients’ stage of rehabilitation, functional status and resources. However, according to participants, an important precondition for successful implementation is a stable financial structure (during and after implementation). They consider that this might be a challenge, considering the history of change regarding the fractioned financing of rehabilitation after stroke in the Netherlands.

## Discussion

The professionals’ perspectives revealed client-centered, caregiver-centered and content specific elements influencing the implementation of HBSR in the Dutch health care system. From a professional perspective important preconditions, such as stroke expertise, transparency of expertise, and professional collaboration, are mentioned as being of paramount importance to implement HBSR. With respect to professional expertise they reported the importance of being educated and actively involved in treating clients after stroke. Regarding professional collaboration, participants mentioned the importance of structured communication facilities and a ‘key agent’ to align interventions. Also, a fitting financial structure is experienced to be essential in order to implement HBSR.

Our study reveals that professionals view the more flexible structure of HBSR, compared to institution-based rehabilitation, to facilitate client- and caregiver-centered treatment. This important asset of HBSR is supported by other studies, recognizing the large heterogeneity in the capabilities of people after stroke [33–36]. Cott [37] underlines that ‘client-centered rehabilitation’ reflects the needs of individuals and groups of clients. It promotes respectful and collaborative partnership between clients and professionals and is recognized as being vital to develop high quality, safe and effective care [38]. A client-centered organization is needed in order to deliver client-centered rehabilitation. According to Epstein et al., client-centered organizations are able to adapt to the often unexpected and context-dependent requirement of situations, rather than relying on fixed protocols [39].

Our study reveals that making use of context-dependent training can be an important considering (potential) cognitive deficits after stroke, while during institution-based rehabilitation there is a gap between training and application setting [40, 41]. Professional perspectives reveal that this context-dependent training can make HBSR more intensive for clients compared to institution-based rehabilitation. This could make HBSR less suitable for clients in frail health. However, clients with higher capabilities could benefit from the higher intensity of HBSR.

Even though our and previous studies have shown positive effects of HBSR, it is not automatically implemented into practice [21, 24–26]. There is a need to develop context specific implementation strategies at different levels [25].

To develop implementation strategies for HBSR, the Consolidated Framework for Implementation Research (CFIR), [25] recommended to pay close attention to factors influenced by innovation complexity. The more informal and loose structure of HBSR and the diversity in context-dependent requirement of home situations makes the implementation of HBSR complex. This could negatively influence implementation, because of the complex context, patient group and collaboration, and the time needed to become competent in ‘delivering’ the client-centered intervention [25]. On the other hand, complex behavioral change interventions can also work in favor of implementation. According to the CFIR, professionals are more likely to do what it takes to fully and effectively implement the intervention if they embrace the intervention as a fundamental change, compared to sites which viewed the intervention as a simple “plug-in” [25]. This fits with our findings of professionals’ experiencing the structure of the HBSR process to be very different compared to institution-based rehabilitation. Its less rigid structure makes HBSR far from a simple “plug-in” intervention. Professionals experience that the rehabilitation process can and needs to be adapted to the (heterogeneous) needs and preferences of clients and caregivers.

Our study also reveals that participants have positive beliefs regarding the relative advantage and (cost) effectiveness of HBSR compared to other stroke rehabilitation interventions. Our study shows that the participating professionals internally developed HBSR as a positive innovation. The large amount of evidence supports their beliefs that HBSR will have the desired outcomes [7–15] and future efforts to implement HBSR is therefore of paramount importance.

With respect to professional views of caregivers needs our study reveals that HBSR might put a high burden on caregivers. As mentioned earlier, professionals experience that the context-dependent and around the clock training makes HBSR more intensive compared to institution-based rehabilitation. But, within the home situation less professional supervision is available. This could imply higher burden on primary caregivers, which highlights the importance of guiding primary caregivers during HBSR. Other studies show that treating clients at home can be a burden on caregivers [42–44]. Meanwhile, caregivers do not always seem to be sufficiently supported, leading to a greater risk of burnout and negative psychological impact [44]. A systematic review by Camak has shown that caregivers have extensive information and educational needs but communication with healthcare providers and/or providing information sometimes seems to be lacking [44]. Also, standardized mostly written information is used [44]. Sit and colleagues [43] underline the importance of tailored information, providing information specific to caregivers’ needs and contexts. To develop implementation strategies for HBSR, according the CFIR [25], close attention should be given to factors influenced by client resources. The presence of a primary caregiver can be a facilitator delivering HBSR but can also be a barrier. When implementing HBSR it is important that involved disciplines sufficiently support the clients’ primary caregiver(s), providing tailored information [7, 10, 45–47]. The recognized more flexible structure of HBSR, facilitating client- and caregiver-centered treatment, may contribute to this.

Our study reveals that participants experience the expertise of primary care professionals to sometimes be insufficient. Participants underline the importance of being educated in the field of neurology and treating clients after stroke on a regular basis. Beaulieu and colleagues [48] underline the importance of professionals’ expertise in order to deliver high quality primary care. They suggest making use of ‘competence-maintenance mechanisms’ (continuous professional development activities and chart audits) to develop and maintain the expertise of primary care professionals [48].

Furthermore our study reveals that professional collaboration needs to be facilitated within primary care because (formal and informal) inter-professional meetings are absent or too irregular. Structured communication facilities and a ‘key agent’ are viewed as supportive factors to inter-professional collaboration. According to Supper and colleagues, inter-professional collaboration within primary care can be defined as “an integrative cooperation of different health professionals, blending complementary competences and skills, making possible the best use of resources” [49]. However, to be able to blend complementary competences and skills, the expertise of professionals should be present and transparent to other professionals. Supper and colleagues confirm that awareness of one another’s roles and competences, shared information and responsibility, and team building are the challenges of interprofessional collaboration within primary care [49]. Establishing a team vision and shared goals, formal quality indicators, information systems, and professionals feeling part of a team could facilitate inter-professional collaboration within primary care [50]. CFIR underlines that, collaboratives and external networking (cosmopolitanism), facilitates the implementation of an innovation. Also, ‘social capital’ including trust, shared vision and information sharing, is considered essential [25]. However, our study indicates that these elements (collaboratives, cosmopolitanism and social capital) are experienced to be scarce within primary care. Therefore, actions to enhance inter-professional collaboration, facing these challenges, should be one of the main parts of implementing HBSR. Our study did not discover knowledge concerning the existence and amount of peer-pressure to implement HBSR. It is important to explore this topic in the future because there is strong evidence that the (absence of) pressure to adopt an intervention, influences organizational adoption and implementation [25].

With respect to financing our study reveals that professionals identify the predefined and restricted nature of the financial structure of HBSR as inappropriate considering the heterogeneity of stroke clients. A systematic review of multi-disciplinary rehabilitation emphasizes that different interventions and combinations of interventions are required because of the diversity in the needs of clients with different problems [36]. Rehabilitation processes are experienced to differ greatly in the professional makeup of the inter-professional teams, organization of services, intensity of therapies, and length of services offered [51]. So, the current financial structure, predefining which professionals and how many hours each can be involved, could hinder the implementation of HBSR. The CFIR emphasizes that policies and incentives have an influence on the implementation of an innovation [25]. Our study also reveals incentives that could facilitate the implementation of HBSR. Institution-based rehabilitation is experienced to be downsized because of the lower (cost) effectiveness compared to other types of rehabilitation. But, in order to implement HBSR, policy makers should face the challenge regarding the financial structure of HBSR and develop a more client-centered way of financing.

## Strengths and limitations

To maximize the credibility of the study, participants varied regarding professional disciplines, workplace settings, and regions. Because all participants, including the focus group moderator (CD), were familiar and interested in HBSR, different professionals perspectives and points of view could be collected. Using a focus group to collect these perspectives could risk opinion contamination among participants. However, the results of our study show that participants reported their thoughts, even if this opposed the views of other participants or even could have been interpreted as questioning the others’ expertise. Another potential limitation of this study can be found in CD (the focus group moderator) being an expert in HBSR and implementation science. Being an expert could lead to the focus group moderator only asking about factors that are known. To prevent this an open ended topic list was used during the focus groups. Also, the focus groups were ended with the secretary providing a brief summary and the focus group moderator asking if anything was missed. CD’s experiences in leading focus groups, facilitated being open-minded and encouraging participants to elaborate on things that they themselves bring forward.

Unfortunately, the perspective of a rehabilitation physician is lacking. Because of the design, study findings might not be transferable to other professionals. However, the results of our study resemble the findings of related literature. Our study appears to have filtered the most important elements from a professionals’ perspectives relating the subject (the implementation of HBSR). Another limitation of this study is the absence of the clients’ and caregivers’ perspectives on factors influencing the implementation of HBSR. It is of paramount importance to collect their perspectives as well. Firstly, because previous study shows that the perspectives of clients’ and caregivers’ can differ greatly from the perspective of professionals [52]. Secondly, previous study shows the complexity of the interaction between clients and their social environment [53]. These topics viewed from a clients and social environment perspective should therefore be studied as well and taken into account when implementing HBSR, specifically because integrated care involves the integration of the perspectives of all stakeholders.

## Conclusion

This study gives a deeper insight in the barriers and facilitators in implementing the complex intervention HBSR within Dutch stroke care from a professional’s perspective. It shows the strength of the intervention and the positive beliefs of the professionals. It also shows the complexity of the outer and inner setting and the process difficulties. Although the Dutch government stimulates the shift from ‘institution-based’ to ‘home-based’ stroke rehabilitation, involved professionals are not (yet) facilitated with information on how this could be done. Currently projects are initiated in response to professionals’ request, to develop the elements that are required to implement HBSR (e.g. prevention of burden of primary caregiver, interprofessional collaboration, better transfer from institution to primary care) [17–19].

Our study suggests that, in order to implement HBSR in daily practice, region specific implementation strategies need to be developed. In line with previous studies [24–26], we suggest developing region specific strategies because ‘contextual confounders’ affect the implementation processes. For instance, ‘degree of collaboration’ can vary between local settings and thereby influences the appropriateness of certain implementation strategies. We recommend developing strategies relating: organized and coordinated inter-professional collaboration, transparency of the expertise of primary care professionals, and the financial structures of HBSR. Further research should be conducted to develop these strategies to a local setting. This should be done in close collaboration with local stakeholders such as client and caregivers organisations, healthcare organizations, primary care professionals and health insurance companies.

## Supporting information

S1 Table Factors influencing the implementation of HBSR in the Netherlands. (DOCX)

S1 File Interview guide focus group (Dutch). (DOCX)

S2 File Interview guide focus group (English). (DOCX)
