# Supplementary material for: Factors influencing the implementation of Home-Based Stroke Rehabilitation: Professionals’ perspective
Source: PLoS One. 2019 Jul 25;14(7):e0220226. doi: 10.1371/journal.pone.0220226 (PMC6657875; doi:10.1371/journal.pone.0220226)
Supplement: S1 Table — (DOCX) [file pone.0220226.s001.docx]

S1 Table: Factors influencing the implementation of HBSR in the Netherlands

| **Determinants of the innovation** | | |
| --- | --- | --- |
| Facilitators | Client-centered rehabilitation process | Opportunity to develop a personalized rehabilitation process based on the clients’ needs, goals and capabilities |
|  | Developing and/or making use of self-management | Developing and/or making use of the self-management capacity of clients by individual support from professionals |
|  | Treatment within clients own environment | Problems generalizing and transferring learned skills from training setting are overcome.  Avoidance of gap between institutionalized setting (with structured and low stimulus environment) and home situation. |
|  | More intensive treatment | Meaningful task specific training “around the clock”. Beneficial for clients with higher (physical and mental) capabilities. |
| Barriers | More intensive treatment | High intensity of treatment makes HBSR less suitable to clients with lower capabilities and/or in frail health. |
|  | Less professional supervision | Some clients need more intensive medical care because of frail health. Some need more supervised training to acquire and maintain skills (e.g. because of reduced learning speed). |
| **Determinants of the user** | | |
| Facilitators | Heterogeneous group of clients | More feasible within HBSR to personally adapt the rehabilitation process to the clients’ situation and capabilities than within (more formally structured) institutionalized rehabilitation. |
| Barriers | Could increase primary caregiver’s burden | Home treatment may be more demanding to the clients’ primary caregiver than institutionalized care. |
|  | Expertise of primary healthcare professionals | Participants experience a lack of knowledge about neurology and phases of rehabilitation among primary healthcare professionals. |
|  | Amount of knowledge and expertise is not-visible | Knowledge and expertise of professionals is not visible. This makes it difficult for clients, caregivers, and referring professionals to know which professional to consult for HBSR. |
| **Determinants of the organization** | | |
| Facilitators | Digital tools are available | Digital tools (e.g. health records and “a collaboration environment”) can facilitate interdisciplinary collaboration within primary care. |
| Barriers | Timeframe screening | Within a short period of time, appropriate care after hospital admission is determined. Some people need some extra time to be suitable for HBSR. |
|  | Lack of (central) coordination of community care | There is a lack of (central) coordination of community care. There should be one “key agent”, who facilitates the client during the different stages of HBSR. |
|  | Lack of interdisciplinary collaboration | Lack of collaboration within primary care because of the large number of different professionals involved in community care.  Also the method and way of documenting treatment findings into health records obstructs interdisciplinary collaboration.  There are no routinely scheduled interdisciplinary meetings within primary care. It takes a lot of effort to organize these when a formal structure of care is absent. |
| **Determinants of the social-political context** | | |
| Facilitators | Creation of stroke-services | Concentrating stroke clients within stroke-services and creating specialized teams could reduce commercial competition and can be supportive regarding development and preservation of expertise and experience. |
| Barriers | Unstable financial structure | For the last years many changes were made concerning the way rehabilitation after stroke is financed. |
|  | Financial structure is predefined and restricted | The way in which HBSR is financed is inappropriate considering the heterogeneity among stroke clients. Involved disciplines and the amount of financed treatment hours are predefined and restricted. |
|  | Temporary assistive devices are not financed | Temporary assistive devices are not financed within the home environment. This may imply high cost for the client which may be a barrier in using the devices and prevent HBSR. |
|  | Commercial competition | Commercial competition between professionals sometimes holds back organized collaboration within primary healthcare. |
