# Supplementary material for: Factors influencing the implementation of Home-Based Stroke Rehabilitation: Professionals’ perspective
Source: PLoS One. 2019 Jul 25;14(7):e0220226. doi: 10.1371/journal.pone.0220226 (PMC6657875; doi:10.1371/journal.pone.0220226)
Supplement: S1 File — (DOCX) [file pone.0220226.s002.docx]

**Interviewguide focusgroep**

*Implementatie van CVA-thuisrevalidatie*

1. **Introductie focusgroep (maximaal 45 minuten)**

Welkomstwoord

*‘’Bedankt dat u tijd vrij hebt kunnen maken om deel te nemen aan deze bijeenkomst’’.*

Introductie

*“De meeste van ons kennen elkaar niet, daarom zou ik willen beginnen met een voorstelrondje.”*

- Eerst onszelf voorstellen (naam, functie, organisatie): eerst voorzitter dan notulist.
- Dan vragen of de deelnemers aan de focusgroep zichzelf willen voorstellen a.d.h.v. naam, functie, instelling en aantal jaren werkzaam, ervaring met thuisrevalidatie.
- Voorstel om elkaar te tutoyeren tijdens de focusgroep en of hier bezwaar tegen is.

Introductie methode

- Bijeenkomst zal 2,5 uur duren, tussentijds nog pauze voor wat te drinken

- Doel van de bijeenkomst: identificeren en bespreken factoren (bevorderend en belemmerend) van invloed op de implementatie van CVA-thuisrevalidatie.

- Uitleg over rollen tijdens de bijeenkomst: voorzitter en notulist

- Uitleg over ‘focusgroep methodiek’: o.a. meningen kunnen verschillen, er is geen goed of fout.

1. **Start focusgroep**

- Definitie CVA-thuisrevalidatie

*Thuisrevalidatie kan omschreven worden als: revalidatie die plaatsvind in de thuisomgeving van de cliënt en die begeleid wordt door 1^e^ lijns professionals. De thuisrevalidatie dient als vervanging van intramurale revalidatie na de acute fase van de CVA. Daarnaast kan de thuisrevalidatie als doel hebben om de lengte van het ziekenhuisverblijf te verkorten. Welke professionals deel uitmaken van het eerstelijns team verschilt per patiënt en is afhankelijk van ervaren beperkingen en de hulpvraag.”*

- Carola presenteert inzichten uit voorgaand onderzoek m.b.t. CVA-thuisrevalidatie en implementatie van innovaties. Conclusie: “v*erschillende onderzoeken hebben aangetoond dat thuisrevalidatie een positief effect kan hebben op cliënt uitkomsten. Dat klinkt allemaal heel positief. Echter in de praktijk wordt ook nog veel revalidatie intramuraal verzorgt.*

*-* Uitleg doel onderzoek:

1. *Inzicht krijgen in de manier waarop CVA revalidatie momenteel wordt aangeboden.*
2. *Het inventariseren van de belemmerende en bevorderende factoren die professionals in de dagelijkse praktijk ervaren met betrekking tot thuisrevalidatie van CVA cliënten.*

- Toestemming vragen voor opname bijeenkomst (incl. uitleg dataopslag en anoniem verwerken).

*Deel A*

*Ervaringen deelnemers m.b.t. CVA-revalidatie (15-20 minuten)*

**Doel**: informatie inwinnen over hoe thuisrevalidatie momenteel in Nederland wordt aangeboden

**Openingsvraag**: *Op welke manier(en) ben jij in de praktijk betrokken bij CVA-(thuis)revalidatie? En wat zijn je ervaringen hiermee?*

**Hulpvragen / hulptopics:**

- Wat was jou rol tijdens dit proces?
- Hoe heb je deze rol ervaren?
  - +: waarom was dit positief?
  - -: welke rol had je liever gehad/Wat was beter geweest? Waarom?
- Wanneer was jij geen onderdeel van thuisrevalidatie van de cliënt?
  - Waarom was je niet betrokken? Had dit anders gekund?
- Wat vind je over het algemeen van de manier waarop thuisrevalidatie aangeboden wordt?
- Met welke disciplines heb je samengewerkt binnen de thuisrevalidatie?

Deel B:

*belemmerende en bevorderende factoren m.b.t. thuisrevalidatie (maximaal 60 minuten)*

**Doel**: Informatie inwinnen over de ervaren belemmerende en bevorderende factoren voor de implementatie van thuisrevalidatie.

**Openingsvraag:** wat zijn factoren die de implementatie van thuisrevalidatie beïnvloeden (zowel positief als negatief)? Je kunt hierbij denken aan factoren m.b.t. de cliënt of mantelzorger, m.b.t. de organisatie, m.b.t. de kennis en vaardigheden van jezelf of andere professionals, m.b.t. het regionale netwerk etc.

**Hulpvragen / hulptopics:** sociaal politieke context (of regionaal niveau), niveau van de individuele organisaties, niveau van de individuele professionals, niveau van de cliënt en mantelzorger (gebruiker).

Tijdens de focusgroep

Alle deelnemers stimuleren tot geven input, ook als ze gereserveerd zijn in het geven van hun mening. Geen nieuwe input meer over een van de topics? Dan afsluiten.

1. **Afsluiting focusgroep (maximaal 10 minuten)**

Notulist noemt belangrijkste conclusies/hoofdpunten die tijdens de focusgroep naar voren zijn gekomen en peilt bij aanwezigen of dit een juiste samenvatting is. Waar nodig vult de voorzitter aan.

Carola bedankt deelnemers voor input en legt vervolgstappen van het projectteam “Thuisrevalidatie” verder uit.
