# Supplementary material for: Factors influencing the implementation of Home-Based Stroke Rehabilitation: Professionals’ perspective
Source: PLoS One. 2019 Jul 25;14(7):e0220226. doi: 10.1371/journal.pone.0220226 (PMC6657875; doi:10.1371/journal.pone.0220226)
Supplement: S2 File — (DOCX) [file pone.0220226.s003.docx]

**Interviewguide focus group**

*The implementation of Home-Based Stroke Rehabilitation*

1. **Introduction (max 45 minutes)**

Welcome

Participants are welcomed with coffee, tea and home-made cake to make them comfortable and to create a loose ambiance. Participants are randomly seated behind tables (set in a square position).

Introductions

- Introduction of focus group leader (name, organization and job role), other present team member (name, organization and job role) and secretary (name, organization and job role).
- Asking permission to tape record the meeting. Information will be used anonymous.
- Participants are asked to name their occupation, work setting, working years and years of experience treating clients within home based (stroke) rehabilitation.
- Propose communicating on first name basis.

Introduction focus group method

- Duration: 2.5 hour (with a break halfway for some more drinks).
- Goal: identifying and discussing factors (facilitators and barriers) influencing the implementation of HBSR.
- Clarification role of focus group leader and secretary.
- Explain focus group technique and endorse people to enlighten their visions (no right or wrong answers, opinions may vary etc.).

1. **Starting the focus group discussion**

- Introducing the subject of discussion by defining “Home-Based Stroke Rehabilitation”.
- Presenting results from prior research concerning HBSR and implementing innovations. Concluding that (although positive outcomes have been established by prior research) rehabilitation in the home environment is not always provided to the clients for whom it could be beneficial.
- Appointing purposes of the focus group: (1) collecting different experiences concerning the way stroke rehabilitation is offered in the Netherlands; (2) determine possible influencing factors (facilitators and barriers) concerning the implementation of HBSR in the Netherlands.
- Ask for verbal informed consent to audiotape the interviews (including why and how data will be handled (saved and assigning ID number to each participant).

*Part A*

*Experiences with stroke rehabilitation (15-20 minutes)*

**Goal**: collecting different experiences in the way HBSR is offered in the Netherlands.

Open-ended questions to start of the conversation:

-> “In what way are/were you involved in stroke rehabilitation?”

-> “What are your experiences?”

**Topics**: positive/negative attitude about that role, preferred role, possible absence of a role, what could/should change, opinion about how HBSR is offered in the Netherlands, collaboration/contact with other disciplines.

*Part B*

*Possible factors (facilitators and barriers) influencing the implementation of HBSR (max 60 minutes)*

**Goal**: collecting different opinions about possible/experienced facilitators and barriers concerning the implementation of HBSR in the Netherlands.

Participants are invited to talk about all kinds of factors influencing HBSR (positive and/or negative) and to freely interact with each other.

**Topics**: factors concerning (1) the social-political context, (2) level of individual organizations (costs and resources, collaboration between professionals, communication, accessibility of professionals, coordination, network size), (3) individual professional (knowledge and expertise, treatment/guidance facilities, involvement, time, motivation) and (4) the user (facilitating personal characteristics, motivation, knowledge/education, satisfaction with delivered treatment, involvement within treatment, medical condition/symptoms).

During the focus group

The focus group leader will stimulate people who seem to be reserved in expressing their opinion by individually asking questions about their experience or opinion.

When saturation is expected to be reached, the focus group leader will evaluated whether every determinant (stated in previous research: determinants of intervention, the user, the organization and the social-political context) is discussed. If necessary, participants will be asked to elaborate some more about specific topics related to the determinants.

1. **Ending the focus group**

Before ending the focus group meeting, the secretary provides a brief summary of the discussion for verification purposes. If participant agree the most important issues concerning the implementation of HBSR are said, the meeting will be ended.

*Official ending:*

- Thanking the participants for participating and their contribution.
- Informing about future steps: data-analysis, reporting back to interested participants (factsheet) and scientific publication of findings (scientific journal).
